# Endobronchial Foreign Body Presenting as Exacerbation of Asthma

**DOI:** 10.1155/2017/6863083

**Published:** 2017-12-13

**Authors:** James E. Tsang, June Sun, Gaik C. Ooi, Kenneth W. Tsang

**Affiliations:** ^1^Royal College of Surgeons in Ireland, Dublin 2, Ireland; ^2^University Department of Medicine, The University of Hong Kong and Queen Mary Hospital, Pok Fu Lam Road, Pok Fu Lam, Hong Kong; ^3^University Department of Diagnostic Radiology, The University of Hong Kong and Queen Mary Hospital, Pok Fu Lam Road, Pok Fu Lam, Hong Kong

## Abstract

Airway foreign bodies are a leading cause of death among children and require urgent recognition by medical personnel. While most cases are diagnosed readily from a clinical history of acute respiratory distress, some cases remain more indolent and present later. We report the case of a 7-year-old boy who aspirated a “LEGO” toy and presented with a week history of increasing respiratory distress compatible with known asthma. Despite a normal chest X-ray, a low-dose computed tomography showed the presence of a foreign body in the left main bronchus, which was subsequently removed by fiberoptic bronchoscopy. Our case serves to reemphasize the importance of considering airway foreign bodies as a cause of respiratory distress, especially in young children.

## 1. Introduction

Accidental aspiration of a foreign body is the 4th most common cause of death among infants and preschool children [1], affecting thousands of children every year, and it remains a common and significant health hazard [2, 3]. In the United States alone, airway FBs are the 3rd commonest cause of death due to unintentional injury in children younger than 1 year [4] and account for more than 17,000 emergency department visits and 220 deaths in children aged 14 years or younger [4]. A prompt and accurate diagnosis, followed by urgent retrieval of the airway FB, is therefore of the utmost importance.

The diagnosis of airway FB is usually obvious with acute onset of dyspnoea, wheezing, and respiratory distress and can be made in 85% of cases at the first physician encounter, as reported in a series of 1269 FB events. However, the remaining 15% of cases can present more elusively, with diagnoses often made after >1 week of delay, leading to complications including pneumonia and atelectasis [5]. Some series report that complications, albeit usually mild, arise in 22–33% of children with airway FB. However, more serious complications such as hydropneumothorax, bronchial stenosis, pulmonary abscess, atelectasis, bronchiectasis, and foreign body dislodgement can also develop, particularly in delayed treatment [6]. Although bronchoscopy is the treatment of choice and usually safe and effective, potentially serious complications occur in 6–8% of procedures, including the development of pneumomediastinum, trachea laceration, vocal cords laceration, subglottic oedema, and necessity for thoracotomy, bronchotomy, or lobectomy [7]. It is therefore imperative not to overlook airway FB among children with respiratory symptoms [8].

We recently encountered a 7-year-old Caucasian asthmatic boy with a 1-week history of subacute deterioration of asthma. He was subsequently found to have a LEGO toy lodged in his left main bronchus. Upon removal of the FB, the patient made a rapid recovery. Our case serves to reemphasize the importance of considering the harbouring of an airway FB in children and adolescents with unexplained respiratory distress.

## 2. Case Presentation

An otherwise healthy 7-year-old Caucasian boy (CS) was admitted as an emergency case on the evening of 23 August 2013 to the Paediatrics Unit of the Hong Kong Adventist Hospital with a 4-day history of increasing respiratory distress. He was well until an incident when he was playing with a LEGO toy at 9 pm four nights before. During this event, CS apparently inhaled a small, round item. He subsequently experienced moderately severe choking and coughing but improved after sleep. The next day, he became wheezy but had no fever or cough. Persistent wheezing led his parents to consult his family physician the day before his admission. CS was prescribed a Salbutamol inhaler for use as required, as the situation was misidentified as a case of asthma, without knowledge of the above incident. He subsequently experienced increasing wheezing and coughing but had no haemoptysis or fever. CS had suffered from regular “bronchitis,” consistent with mild asthma, twice a year since 3 years of age. He, nonetheless, had a clinically uneventful preceding year until the episode in question. He was hospitalised briefly with an episode of community-acquired pneumonia two years beforehand. He was on no regular medication and had no known allergy.

**Figure 1:**
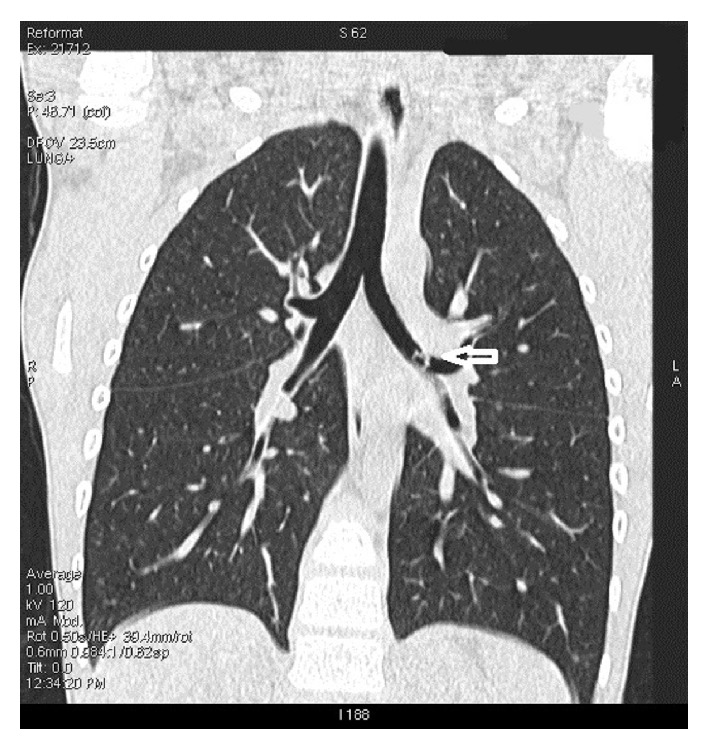
A low-dose CT thorax, at coronal plane, showing an intraluminal foreign body with an inverted U appearance (arrow) lodged at the distal left main bronchus measuring 5.4 × 6.8 × 7 mm.

Physical examination initially showed a well-developed boy who was rather uncomfortable. He had hoarseness, which was apparently long-standing. His blood pressure was 110/59 mmHg and weight was 23.5 kg. He had a fever of 37.6°C and a room air SaO2 of 95%. Examination of his chest showed isolated left-sided wheezing and reduced breath sounds, but otherwise normal for respiratory examination. He had no cervical lymphadenopathy or finger clubbing. There was no surgical emphysema. Physical examination was otherwise normal for the cardiovascular and abdominal systems. His chest X-rays taken one day before admission, by his family physician, and upon emergency admission were both normal. He, therefore, underwent a low-dose CT thorax, which showed an intraluminal foreign body with an inverted U appearance lodged at the distal left main bronchus measuring 5.4 × 6.8 × 7 mm (Figure 1).

**Figure 2:**
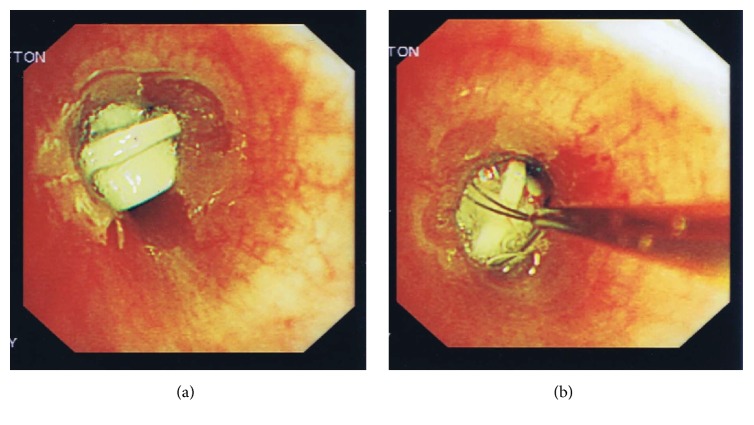
(a) Photograph showing the wedging of a “LEGO” in the left main bronchus of our patient (CS) and surrounding mucosal inflammation. (b) Photograph showing the capture of the “LEGO” in the left main bronchus of our patient (CS) by the Dormia Basket (Gemini).

The patient, therefore, underwent a fiberoptic bronchoscopy, using a standard size Olympus BF F260 adult bronchoscope under local anesthesia and sedation, on the evening of 25 August 2013 with the help of a senior anaesthetist. At bronchoscopy, the upper airway, including the vocal cords, was normal. The entire trachea was normal and carina was sharp. Right main bronchus had mildly copious amount of mucus and the left main bronchus had moderate mucosal inflammation with mucus stagnation. There was a foreign body, with similar morphology revealed by the CT thorax, wedged firmly in the proximal left main bronchus with surrounding mild mucosal inflammation and haemorrhage (Figure 2(a)). A Paired Wire Helical Stone Retrieval Basket (Germini™, Boston Scientific) was, therefore, passed via the suction channel of the bronchoscope and was able to grip tightly around the FB. The FB was subsequently removed with withdrawal of the bronchoscope after the first attempt (Figure 2(b)). Upon removal of such, the left main bronchus showed slight mucosal haemorrhage, which subsided spontaneously. Subsequent examination of the left upper, lingula, and left lower lobes was normal down to subsegmental level.

CS was, therefore, treated after bronchoscopy with IV fluid, IV Maxipime 1 g BD, and nebulized Salbutamol and Budesonide. He was immediately relieved of his cough and respiratory distress, and his chest became normal on examination, post-op. There was a spike of 38.5°C which lasted for 4 hours post-op. His chest X-ray the day after bronchoscopy was again normal. His chest became clear, and his SaO2 on room air on discharge was 99%. There was no fever. He was discharged on 25th September 2013, 2 days after his emergency admission on Augmentin for three days and Symbicort 80 one puff BD. Upon review one week later at the outpatient clinic, CS reported no respiratory symptoms and repeatedly stated he had become extra cautious with his LEGO and other toys. His chest was clear on examination and there was no cervical lymphadenopathy. He was discharged and has been symptom-free to date.

## 3. Discussion

Asthma is the commonest chronic disease among children, and industrialized countries experience high lifetime asthma prevalence that has increased over recent decades [9]. The diagnosis for asthma is usually obvious clinically, although this is occasionally confused with other conditions, most notably with airway obstruction from other pathology. Asthma is occasionally wrongly diagnosed in the presence of airway FBs both in children and in adults, although this is seldom reported. In one case, a 9-year-old child was misdiagnosed as having asthma, which stemmed from symptoms secondary to stagnation of a tack in the bronchial tree for several years [10]. Occasionally, airway FB presents with clinical features suggestive of late-onset asthma, as reported in a 56-year-old Japanese subject, who even developed physiological evidence of obstructive pulmonary dysfunction and airway hyperresponsiveness to inhaled Methacholine [11]. In our patient, the typical wheezing and dyspnoea in a long-standing history compatible with asthma made it difficult to initially appreciate the presence of an airway FB, until intensive cross-examination of the patient and parents. This case serves to reemphasize the need to actively exclude airway FB among children, especially those under three years of age, with unexplained respiratory distress [5].

The risk factors for airway foreign bodies in adults include psychiatric and neurological disorders, severe trauma, alcoholism, sedative usage, poor dentition, and advanced age [12]. Such clear stereotyping has not been identified among children with inhalation of airway FB, but such frequent occurrence is attributed to young children's tendency to suckle and store foreign materials including foodstuffs in their mouths, alongside a proclivity for simultaneous running and crying. Paediatric airway foreign bodies are more common among children <3 years of age [5], boys [13], and nonwhites [14], compared with their counterparts. A large variety of airway FBs have been reported, including food (75%) and other organic materials such as plants (7%), inorganic materials such as metal and plastic objects (14%), and less commonly toys or parts of toys (1%) [15, 16]. These are predominantly located in the right bronchial tree (48-49%), less in the left (39–44%), and least likely in the upper airway or tracheal (4–13%) [15, 16]. Younger children appear to have a higher tendency to inhale organic food material while their counterparts inhale inorganic materials [17]. One series on 165 children showed that conforming objects such as balloons caused significantly more deaths in those ≥3 years than their counterparts and accounted for 29% of all deaths [14].

Inhalation of FB into the airways may be witnessed. Presence of a witness for the inhalation incident is usually diagnostic, although this does not guarantee the presence of an airway FB in situ as the patient can dislodge or cause migration of the airway FB through intensive coughing. There is a clear need for an early diagnosis for patients with airway FB although it is not always easy to diagnose airway foreign body, especially as young children under the age of three often cannot present a clear story. About 5.3% of the cases present 4–12 weeks after the aspiration incident [18] and thus present challenges in the diagnosis. The presence of choking, wheezing, and coughing occurs in 47.9–95% of cases [13, 17–19], but these symptoms are common and often nonspecific among children. In contrast, acute or recurrent infection was the most frequent clinical presentation among adults with airway FB, which is uncommon for the general adult population [20]. The presence of an abnormal chest X-ray, including ipsilateral hyperinflation, atelectasis, infiltration or frank consolidation, and at later stages bronchiectasis, occurs in 42–73% of patients. A normal chest X-ray occurs in 33–52.4% of patients [21]. The use of inspiratory and expiratory views or fluoroscopy, to demonstrate air trapping or mediastinal shift, is not sensitive and nondiagnostic [19, 20, 22, 23]. Magnetic resonance imaging with T1-weighted images can also be useful for the diagnosis and location of peanut fragments in the lower airway [24], but the long scanning time, poor visualization of lung parenchyma, and the potential claustrophobia present challenges in an unwell child. Low-dose computed tomography (CT) is the most appropriate imaging modality, especially before proceeding to bronchoscopy, in light of its high speed, clear airway, and lung parenchymal resolution and availability [25]. Among 45 consecutive children with suspected FB aspiration, low-dose CT thorax identified 100% of the FBs, and its negative findings for such prevented 3 patients from proceeding to bronchoscopy [26]. Fiberoptic bronchoscopy is generally considered as the gold standard of diagnosis, as it will permit direct visualization of the major airways where FBs are often lodged [1]. More recently, the use of virtual CT bronchoscopy has also been proposed to examine the airways in the event of a suspected FB in situ and to help plan the bronchoscopic procedure [27].

Before the advent of bronchoscopic techniques, the mortality rate for airway FBs was unacceptably high at around 50% [28]. Many clinicians advocate the use of rigid bronchoscopy, performed under general anesthesia, as the standard FB extraction procedure [29]. The use of such, however, is associated with morbidity and mortalities. In one series, 5 patients (0.6%) died after the bronchoscopic procedures [13], whether or not it was possible to directly attribute to the rigid bronchoscopy, general anesthesia, or individual patient parameters. Flexible bronchoscopy is generally regarded as the first-line procedure to remove airway FB in adult patients [30]. With increasing experience and development of better accessories, removal using a flexible bronchoscope under local anesthesia can be performed safely and successfully, as demonstrated in our case. A recent review of a series of 400 cases showed a success rate of 86% using flexible bronchoscopy [31]. Some authors also advocate proceeding directly to bronchoscopy, even with a negative chest X-ray, if there is clinical suspicion of an airway FB in children [13, 19].

Foreign body inhalation is not uncommon in children and bronchoscopy should be performed at the earliest opportunity when there is suspicion of foreign body inhalation, even in the case of a negative chest radiograph, preferably after an urgent low-dose CT thorax.
